# *Arcanobacterium haemolyticum *associated with pyothorax: case report

**DOI:** 10.1186/1471-2334-5-68

**Published:** 2005-09-06

**Authors:** Subhash Chandra Parija, Venkatesh Kaliaperumal, Saka Vinod Kumar, Sistla Sujatha, Venkateshwara Babu, V Balu

**Affiliations:** 1 Departments of Microbiology, Jawaharlal Institute of Post-graduate Medical Education and Research, Pondicherry, India; 2 Tuberculosis and Chest Diseases, Jawaharlal Institute of Post-graduate Medical Education and Research, Pondicherry, India; 3 Government hospital for chest diseases, Gorimedu, Pondicherry, India

## Abstract

*Arcanobacterium haemolyticum *has an established role in the etiology of human pharyngitis. There are increasing reports of systemic infections caused by this organism. From India, we report the first case of *Arcanobacterium haemolyticum *causing pyothorax in an immunocompetent adolescent male patient. The probable mode of infection is also discussed. The role of *A. hemolyticum *as an animal pathogen needs further study.

## Background

There are many reports of pharyngitis and wound infections associated with *A. haemolyticum*. Rarely systemic infections have been documented [1,2]. Most of these infections tend to occur in the adolescent age group. It has been isolated from symptomatic individuals, some times as a co-infection along with other pathogens [3,4]. Human beings are thought to be the reservoir for this organism. But there are few reports of animal infection caused by this organism [5,6]. The role of *A. haemolyticum *as a zoonotic disease and its differentiation from the closely related animal pathogen *Arcanobacterium pyogenes *needs to be defined [7-10]. *A. pyogenes *is one of the agents of bovine mastitis and is also known to cause pyogenic infections in human [11,12]. To our knowledge this is the first report of pyothorax associated with *A. haemolyticum *from India. A similar case of pyothorax and septicemia by this organism has been documented in United Kingdom by Stacey and Bradlow in 1999 [4].

## Case presentation

A 19 year old male was admitted to the department of tuberculosis and chest diseases, with complaints of gradually progressive breathlessness of one week duration along with low grade fever and cough with foul smelling expectoration. Patient was not on any antimicrobial treatment. There was no history of trauma to the chest and his past medical history was unremarkable. He did not have pharyngitis or skin rashes. He was a non-smoker and non-alcoholic and was employed in a tea stall, wherein he handles unpasteurized milk.

**Figure 1:**
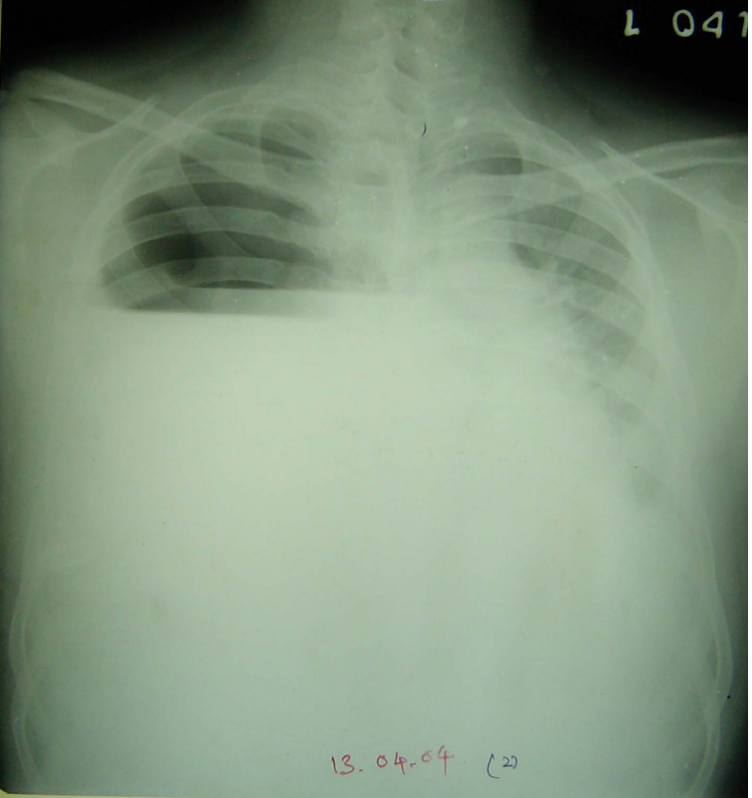


Clinical examination and imaging studies revealed a right sided multi-loculated hydropneumothorax (Figure 1). Blood peripheral smear, total and differential leukocyte counts were normal. Routine serum biochemical parameters were also within normal limits. ELISA test for HIV was negative.

**Figure 2:**
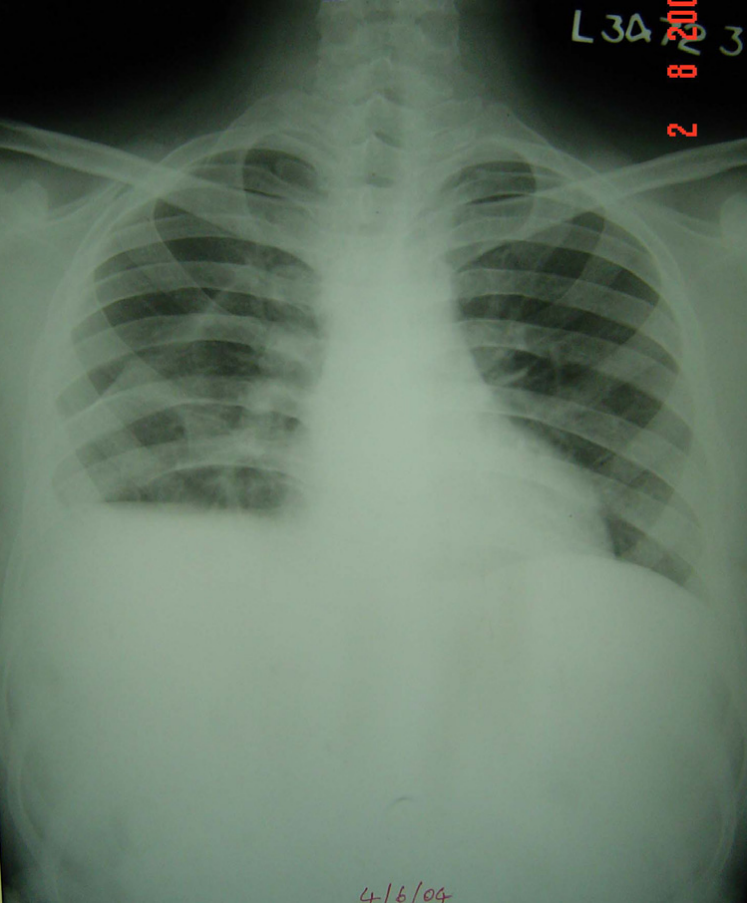


Therapeutic thoracocentesis yielded 500 ml of thick foul smelling pus and air. Empirical treatment with intravenous ceftriaxone and metronidazole was started. Patient was put on closed chest tube drainage with underwater seal which was removed after one month, in view of significant clinical and radiological improvement (Figure 2). Antimicrobial therapy was also continued for the same duration. Patient was then discharged and advised follow-up.

**Figure 3:**
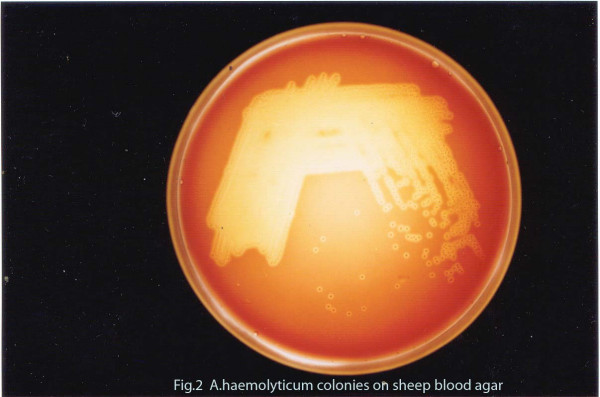


**Figure 4:**
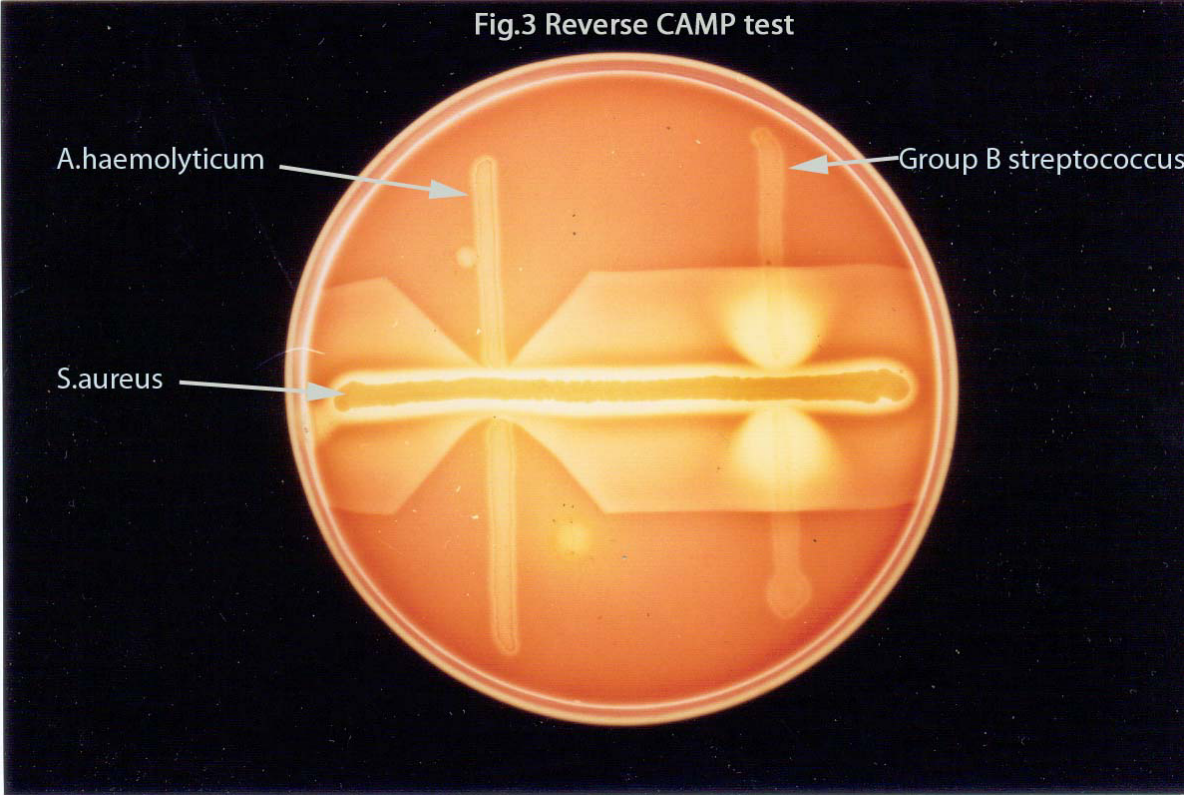


Microscopic observation of the Gram stained smear of the pus showed pleomorphic Gram positive coryneform bacteria. Aerobic culture after 48 hours at 37°C grew minute, translucent, non-pigmented colonies with a small zone of clear hemolysis on 5% sheep blood agar (Figure 3). Heavy growth was obtained in pure culture. Upon further incubating the culture plates the colonies increased in size and appeared opaque with enhanced hemolysis. Transmitted light revealed a small dark dot at the centre of each colony. When a colony was gently removed a tiny dark pit was seen in the agar. The isolate did not grow in MacConkey agar. It was non-motile, non-acid fast and did not show any volutin granules. Catalase and urease tests were negative. Glucose and maltose were fermented but not xylose and mannitol. Reverse CAMP (Figure 4) and DNAase tests were positive. Gelatin was not liquefied.

Based on the hemolytic pattern, catalase reaction and other biochemical tests the isolate was identified as *Arcanobacterium haemolyticum*. These tests also differentiate our isolate from the closely related *Arcanobacterium pyogenes *[8]. The isolate was found to be sensitive to routinely used antibiotics (Ampicillin, 10 mcg/disc ; erythromycin, 15 mcg/disc ; ceftriaxone, 30 mcg/disc; gentamicin, 10 mcg/disc and ciprofloxacin, 5 mcg/disc) by Kirby-Bauer disc diffusion method done on Muller Hinton Agar supplemented with 5% sheep blood.

## Conclusion

Our isolate from a sterile site and in pure growth confirms the pathogenicity of this organism. Often *A. haemolyticum *is overlooked as normal flora as the hemolytic pattern is not pronounced on primary isolation after 24 hours of aerobic incubation [7].

Since identification of all the coryneform isolates is difficult, the source and mode of infection of many pathogenic coryneform bacteria remains obscure. We have identified our isolate based on its phenotypic characters. An interesting observation from this case and from another case of pharyngitis is that one of the risk factor for the infection could be close contact with animals like cows and buffaloes and handling or consumption of unpasteurized milk. In this case of pyothorax, the probable mode of infection could be occupation related exposure to aerosols from unpasteurized milk. **This hypothesis, however, is merely speculative and needs confirmation**. Detailed studies regarding the exact identification of the coryneform isolates and their importance in human and animal infection with regards to the environmental and geographic variables is warranted.

In summary, while not frequently recovered from clinical specimens, *A. haemolyticum *may cause serious systemic infections which need to be distinguished from closely related *A. pyogenes*. The source and the mode of infection need to be investigated for taking appropriate preventive measures.

## Authors' contributions

SCP, SS and VK had isolated and identified the organism and have made substantial contribution for drafting the article. Balu, Babu and SVK were the physicians involved in treating the patient. SS and VK had isolated and identified the organism.

## Pre-publication history

The pre-publication history for this paper can be accessed here:


